# *Rhynchophorus palmarum* in Disguise: Undescribed Polymorphism in the “Black” Palm Weevil

**DOI:** 10.1371/journal.pone.0143210

**Published:** 2015-12-18

**Authors:** Bernhard Löhr, Aymer Andrés Vásquez-Ordóñez, Luis Augusto Becerra Lopez-Lavalle

**Affiliations:** 1 Corporación Colombiana de Investigación Agropecuaria, Centro de Investigación Palmira, Palmira, Valle del Cauca, Colombia; 2 Entomology Program, Centro Internacional de Agricultura Tropical, AA 6713, Cali, Valle del Cauca, Colombia; 3 Cassava Program, Centro Internacional de Agricultura Tropical, AA 6713, Cali, Valle del Cauca, Colombia; Ecole des Mines d'Alès, FRANCE

## Abstract

During studies to adapt pheromone trapping of *Rhynchophorus palmarum* to the special coconut growing conditions at the Colombian Pacific coast, 152 atypically-colored specimens were captured in a total collection of 53,802 of the normally completely black weevil. Five specimens had the typical coloration of *Rhynchophorus ferrugineus*, an invasive species recently introduced to Aruba and Curação. A regional expansion of this invasion to the South American continent was feared and all atypical specimens were submitted to taxonomic analysis. Both conventional and molecular methods were employed. Conventional taxonomics confirmed the samples as belonging to *R*. *palmarum* but registered undescribed and species-atypical morphological variability in the subgular suture (wide vs. narrow), the ratio between intraocular distance and width of antennal scrobes (>0.35 vs. < 0.29) and the indentation of the mandibles (up to three mandibular teeth vs. bilobed). Molecular analysis placed all samples inspected, black and reddish alike, firmly within the *R*. *palmarum* group and the hypothesis of having inter-specific hybrids was rejected using co-dominant single sequence repeat markers with allelic specificity for both species.

## Introduction

The American or black palm weevil (APW), *Rhynchophorus palmarum* (L), is on record as the worst pest of palms in tropical America. It is not only a primary pest of palms, its pest status is greatly aggravated by its vectoring of the nematode, *Bursaphelenchus cocophilus* (Cobb), the cause of the red ring disease (RRD) [1]. The APW/RRD complex has destroyed thousands of coconut palms, the problem being particularly bad in Tumaco Bay at the Colombian Pacific coast, where entire plantations are wiped out every 12–15 years [2].

In an effort to alleviate the problem we tried to adapt the standard method of APW control—mass trapping using aggregation pheromone baited traps—originally developed for coconut and oil palm plantations [3]—to the small-holder coconut production system at the Pacific coast. As APW also occurs on sugarcane, much of the experimentation was conducted at the Centro Internacional de Agricultura Tropical (CIAT) headquarters in the Colombian Cauca Valley, the main sugarcane production area of Colombia, where trapping of weevils was conducted as a routine pest control measure.

**Fig 1:**
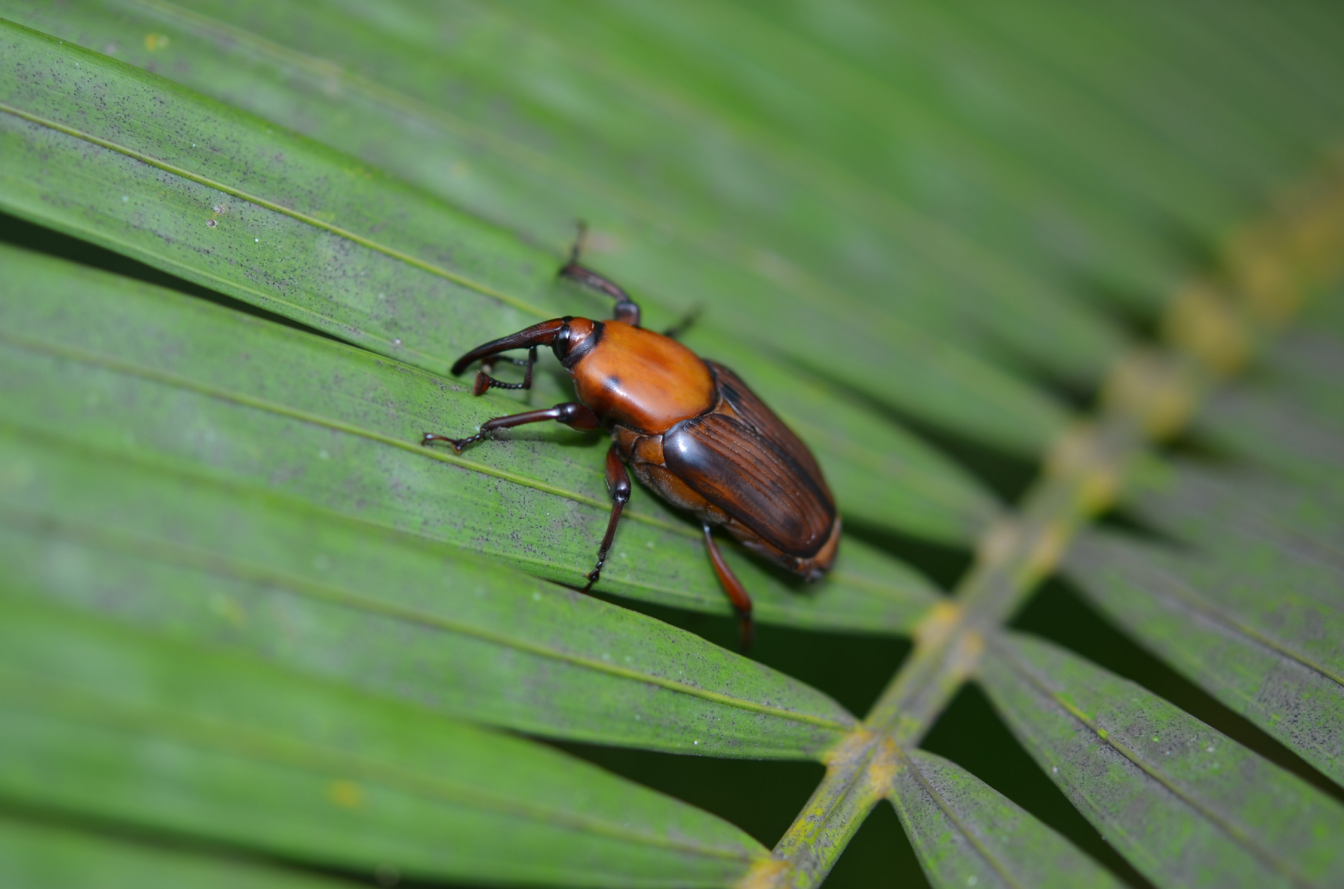
Female *Rhynchophorus palmarum* with coloration resembling *R*. *ferrugineus*, captured in a sugarcane plantation at CIAT headquarters, Palmira, Colombia, on February 25, 2014.

In the course of these activities, over 50,000 weevils were captured during a period of roughly one year. All weevils were sexed and thus closely inspected. On July 27, 2012, an unusual specimen was captured: instead of being completely black, the natural color of APW, the ventral part of the thorax of this specimen was dark red. This was recorded but not much attention was given to it. However, more specimens with aberrant color were caught later on and this increased considerably during the rainy season of 2013. On May 19, 2013, we caught a female with the typical coloration of the red palm weevil, *Rhynchophorus ferrugineus* (Olivier) (Coleoptera: Dryophthoridae) (Fig 1) and two days later a male. The Colombian authorities were informed about the suspected capture of an invasive species and the female was sent to the Instituto Colombiano Agropecuario (ICA) for identification. The specimen was identified as *R*. *palmarum* but since color morphs of this species had never been recorded, doubts as to its identity remained. In view of the importance of commercial oil palm and coconut production in the country, further studies of the phenomenon appeared mandatory.

*Rhynchophorus ferrugineus*, the red or Asian palm weevil (RPW), has a long history of invading new areas. Originating in humid tropical Asia [4,5], the species has managed to expand its geographical range of occurrence well beyond areas that might seem suitable for the weevil at first glance. In the early eighties it was first recorded outside its natural range of occurrence when it was detected in date palm on the Arabian Peninsula [6], becoming probably the first insect pest from the humid tropics to invade a desert environment and causing huge damage [7]. Roughly at the same time a separate introduction occurred into Iran [8] and from 1993, after its first detection in Spain [9] it spread all over the Mediterranean.

The main pathway seems to have been via the importation of tens of thousands of tons of infested adult date palms, principally from Egypt, for land-scaping projects in new residential developments [10]. However, at that time the weevil was already well-known as a major phytosanitary problem in the Middle East, particularly Egypt. Neither the exporters nor the importers seem to have given much thought to the possible consequences of these shipments. The European Union phytosanitary authorities only reacted with an ordinance in 2007, 14 years after the problem was first detected. By that time, the pest was well established in many Mediterranean countries [11] causing the death of hundreds of thousands of canary date palms, *Phoenix canariensis* Chabaud (Arecaceae), the typical, landscape dominating palm of the Mediterranean.

In spite of this calamity in the Mediterranean and the Middle East, mature date palms were imported–again from Egypt—for landscaping projects in hotel developments on the Caribbean island of Curação [12] (65km from the South American continent) and RPW was detected in December 2008 [13] and in 2009, RPW was detected on another island, Aruba [14], only 26km from the continent. Efforts to eradicate the pest on the islands failed. As the economies of both islands rely heavily on tourism and local food production cannot satisfy demand, much is imported from the continent, resulting in a steady traffic of boats. This together with the failure of eradication and the proximity of the coast of Venezuela is a dangerous combination of factors that could eventually facilitate the spread of the weevil to the South American continent.

The American palm weevil, *R*. *palmarum*, is also known as the black palm weevil and no color variants are on record [4, 15] while *R*. *ferrugineus* is known to be very variable [4, 16], hence we worked with two interconnected hypotheses: 1 we have detected an invasion of *R*. *ferrugineus*, and 2 the unusual color morphs that were captured in the pheromone traps are interspecific hybrids between the local and the invasive palm weevil. We employed conventional and molecular taxonomic methods to determine the identity of the color morphs captured and on the basis of our results, both hypotheses were rejected.

## Material and Methods

### Collection of palm weevils

All palm weevils were captured in traps baited with aggregation pheromone (Rhynchophorol, Laboratorio de Bioproductos, Bogotá, Colombia) of *R*. *palmarum* at CIAT headquarter (coordinates: 3°30'477"N, 76°21'1830"W) in 2012 and 2013. As the trapping was part of routine pest control activities, a permit for collections was not required. The traps consisted of a 20 l white plastic container (25X25X60 cm) with two windows of 12x8cm cut out of the wall on opposite sides on the upper part of the container. A small hole was drilled in the top of the container to introduce a string where the pheromone dispenser was suspended. Two stakes of sugarcane of 25cm length, split in the middle were added as food bait in each trap. This set-up was standard in all traps. The sugarcane was changed as necessary. This could be as often as daily when the weevils were captured alive and sometimes amounted to several hundred in 24 hours so that the sugarcane was completely destroyed or once in four weeks when it was treated with preservative and insecticide. Traps were placed at 80 – 100m between traps and emptied daily in the afternoon and the weevils were sexed and counted.

The traps were part of three experiments, designed to adapt the trapping technology to the special growing conditions at the Pacific coast. The details of these experiments are documented in [17]. All captured weevils were sexed and checked for aberrant color morphs. Weevils with unusual coloration were recorded and kept in the deep freezer for morphological characterization and eventual molecular analysis. Additional specimens were captured manually at CIAT headquarters from March to December 2014. All unusually colored specimens were deposited in the CIAT Arthropod Reference Collection (CIATARC), the black morphs were eliminated after inspection. In addition to these, all *Rhynchophorus* specimens in the entomological collection of the Universidad Nacional de Colombia, Palmira campus (CEUNP) were also studied. The specimens of this collection are from different localities of Cauca and Valle del Cauca Departments of Colombia.

### Morphological characterization of samples

The morphological characterization employed the traditional characters used in the differentiation of *Rhynchophorus* species [4, 18]. Special emphasis was given to characters for differentiating *R*. *palmarum* from *R*. *ferrugineus*, *R*. *phoenicis*, *R*. *bilineatus* and *R*. *vulneratus* (named here as *R*. *ferrugineus* “group” [4]). These characters were: shape of posterior margin of pronotum (produced or extended medially vs. oval or broadly); width of subgenal suture and ventral distance between antennal scrobes (narrow vs. wide); relationship between interocular space and width of rostrum at base (one-third or less vs. not less than one-third); shape of dorsal tip of rostrum (grooved or nearly truncate vs. oval distally).

The distance between antennal scrobes was measured with an ocular micrometer, while the ratio of interocular space to width of rostrum was calculated on basis of the pixel numbers in photographs of these structures using Adobe Photoshop CS4. The presence of all important characters cited for the differentiation of *R*. *palmarum* and *R*. *distinctus* were also confirmed, as both species share the characters mentioned above. An additional character examined and documented was the structure of the aedeagus, following the methods described by Wattanapongsiri [4]. In total, 182 trap-collected specimen (all 152 reddish and a sample of 15 male and 15 female black specimens) and 283 specimens, including one with reddish color, deposited in the CEUNP collection were inspected. Permission to examine the weevils in the collection was granted by the curator, Prof. Nhora Meza.

Color pictures of all characters were taken with a Canon Eos 60 D equipped with a Canon macro lens EFS 60 mm in the MK Photo-eBox Plus 1419^TM^ and with the same camera mounted on a Olympus SZ61 Zoom Stereo Microscope combined with a DL106 High Brightness Diffuse Dome.

### Molecular analysis

For the DNA analysis, the 15 specimens most resembling *R*. *ferrugineus* and a sample of three each of normally colored males and females were selected. The first two legs on the right-hand side of all specimens investigated were removed, labeled and returned to the deep freezer for later DNA extraction. Total genomic DNA was obtained from the specimens using a modified cetyltrimethylammonium bromide method (CTAB) [19] by adding 25 M potassium acetate (pH 55) to precipitate protein. The DNA-barcode region was amplified, cloned and sequenced as described by Ovalle et al. [20].

### DNA sequence analysis

DNA Sequences were edited to the *R*. *palmarum*-specific COI sequence by eliminating vector and universal primer sequence and analyzed for identity with sequences deposited in the GenBank, EMBL or BOLD public databases. COI sequence contigs were assembled to identify unique haplotypes with a discrimination requirement of 100% identity. Genetic distances were calculated with the alignment sequences for six species of the genus *Rhynchophorus*. Further, we used sequences from the sub-families Dryophthorinae, Cerambycinae and Entiminae as outgroups. Three analyses were performed by using the MEGA V 505 software. The first was phylogeny reconstruction using the statistical method UPGMA under the 2-parameter model of base substitution. The second analysis applied the standard neighbor-joining (NJ) K2P model. Finally the data were also analyzed phylogenetically using maximum parsimony (Maximum Likelihood Method). For these analyses, the data were pruned to only unique haplotypes. When the number of common sites was < 100 or less than one fourth of the total number of sites the maximum parsimony method was used; otherwise the BLONJ method with MCL distance matrix was used.

### Microsatellite markers for assessing potential hybridization between *R*. *palmarum* and *R*. *ferrugineus*

Genomic DNA was obtained from 26 palm weevils (21 *R*. *palmarum* and five *R*. *ferrugineus*). Seven *Rhynchophorus*-specific SSR co-dominant makers were selected for assessing potential hybridization between *R*. *palmarum* and *R*. *ferrugineus* (Table 1) [21]. Sequence-specific PCRs were performed essentially as described by Schuelke [22] and Rampling [23] with the following modifications: A M13 (-19) primer was labeled with 6-FAM^TM^, or NED^TM^ covalently bound to the 5’ end for detection on the ABI 3130xl (Applied Biosystems, CA-USA). The unlabeled primers in each reaction consisted of a *Rhynchophorus* SSR-targeting forward primer with a 5’ M13 tail and a SSR-targeting Rev. After PCR amplification, 6-FAM^TM^ and NED^TM^ amplification products were mixed according to their size and labeling. Electropherograms were analyzed using the GeneMapper® version 4.0 software (Applied Biosystems, CA-USA). The number of alleles per locus for each genotype at each of the loci, were organized in a matrix in a Microsoft® Office Excel file for analysis.

**Table 1:** Characteristics of seven microsatellite loci isolated in *Rhynchophorus ferrugineus* (adapted from Capdevielle-Dulac [21]).

ID	Primer type	Primer sequence	Repeat targeted
*P1A3*	M-Fwd (5'–3')	***CACGACGTTGTAAAACGAC***CACCTTTAATAGTTCTTCTGACAT	(GT)_14_
*P1A3*	Rev (5'–3')	AAAAGACAAGGAAATCCACA	(GT)_14_
*P1C11*	M-Fwd (5'–3')	***CACGACGTTGTAAAACGAC***TCCTGCGAACAAAGAGAAA	(TG)_10_
*P1C11*	Rev (5'–3')	GCAAAAATCACTCGGACA	(TG)_10_
*P1E2*	M-Fwd (5'–3')	***CACGACGTTGTAAAACGAC***CATTGATGTTGATTTTCGATT	(GA)_20_
*P1E2*	Rev (5'–3')	ACCATGAGATCGGCTGTTT	(GA)_20_
*P2F11*	M-Fwd (5'–3')	***CACGACGTTGTAAAACGAC***TGACTCATGGATTTTGTCATT	(AG)_10_
*P2F11*	Rev (5'–3')	GGCAACTCTTTCGCACTTT	(AG)_10_
*P2F8*	M-Fwd (5'–3')	***CACGACGTTGTAAAACGAC***GCCTTAGACTTTGTCCTACCC	(TG)_8_
*P2F8*	Rev (5'–3')	ATTCCTTATTCGCCTGACTT	(TG)_8_
*P4C2*	M-Fwd (5'–3')	***CACGACGTTGTAAAACGAC***ACAACATTTTCACCAAATTCA	(TG)_16_
*P4C2*	Rev (5'–3')	TCTTGTTCTTGATAAACCCAACT	(TG)_16_
*P1F8*	M-Fwd (5'–3')	***CACGACGTTGTAAAACGAC***TTAGATGCTACGTGATAGAAGAC	(GA)_27_
*P1F8*	Rev (5'–3')	CAGCCGGTCCATACACA	(GA)_27_

The Excel matrix was used to assess all allele differences between the two species for the construction of the genetic relationship matrix which was employed to unravel any parental relashionship and to present graphically the results.

## Results

Out of a total of 53,802 weevils captured in the traps, 152 (0.28%) were specimens with reddish coloration on at least one part of the body. Similarly, one of the 283 *R*. *palmarum* deposited in the CEUNP collection presented a reddish coloration. This red specimen was collected at Guacarí, Valle del Cauca, (3°45'4808"N, 76°19'5705"W), located 28.8 km north of the location of the trap experiments.

**Fig 2:**
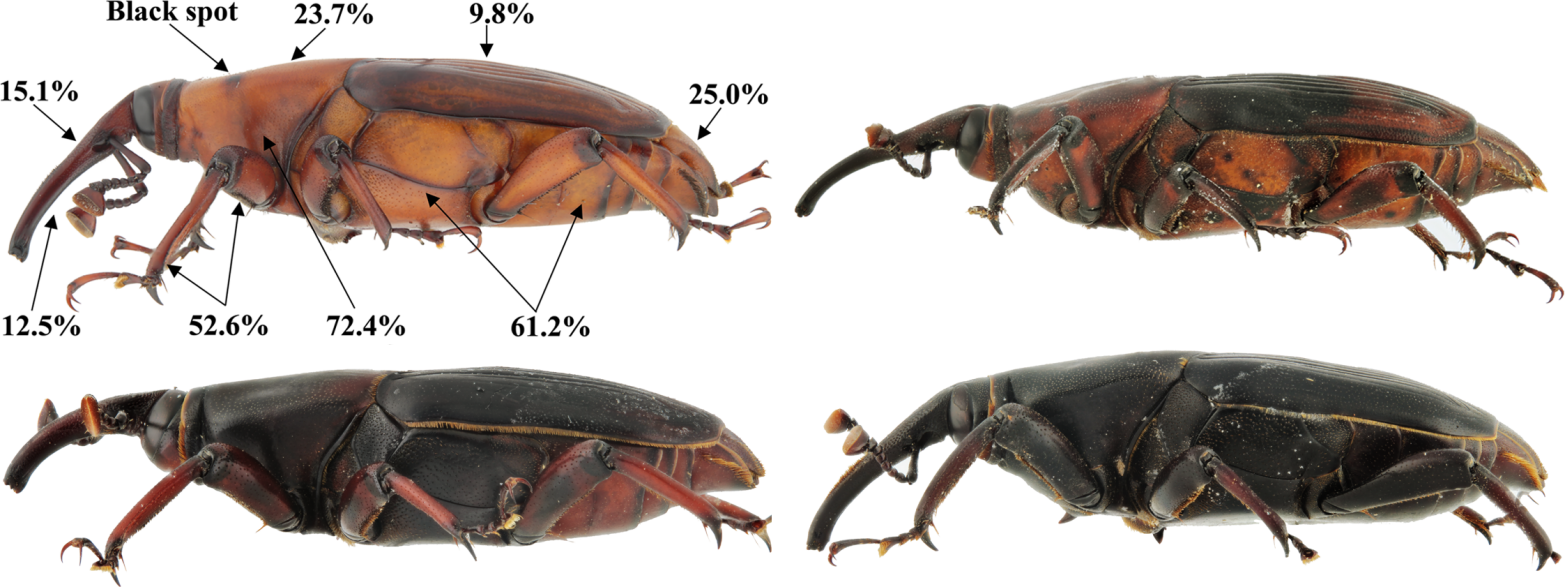
Atypically colored males (top left and bottom) and female (top right) of *Rhynchophorus palmarum* captured in pheromone-baited traps. Percentages indicate the proportion of atypically colored weevils presenting off-color on the different body parts of the naturally all-black weevil. Overall, 152 off-colored specimens were caught in a total collection of 53,802 weevils.

The coloration of the reddish specimens ranged from deep dark red to reddish brown and bright orange (Fig 2). Three specimens, one male and two females showed the typical coloring known so far only from *R*. *ferrugineus*: reddish brown head, thorax and abdomen ventrally, dark red elytra and orange thorax dorsally with two symmetrical lateral dark spots (Fig 1). There was a clear difference in the distribution of the red color with 94.8% of all 152 colored specimens showing red on the underside while only 38.8% presented red on the dorsal part of the body (S1 Database). The lower thorax was the part most frequently red in color (72.4%), followed by the abdomen (61.2%) (Fig 2). The head was the only body part where the off color was more frequent dorsally (15.1%) than ventrally (12.5%) and 9.8% of the specimens presented red-colored elytra. About half of the specimens had colored legs (52.6%). Sex also seems to play a role with only 39.5% of the colored weevils being females.

**Fig 3:**
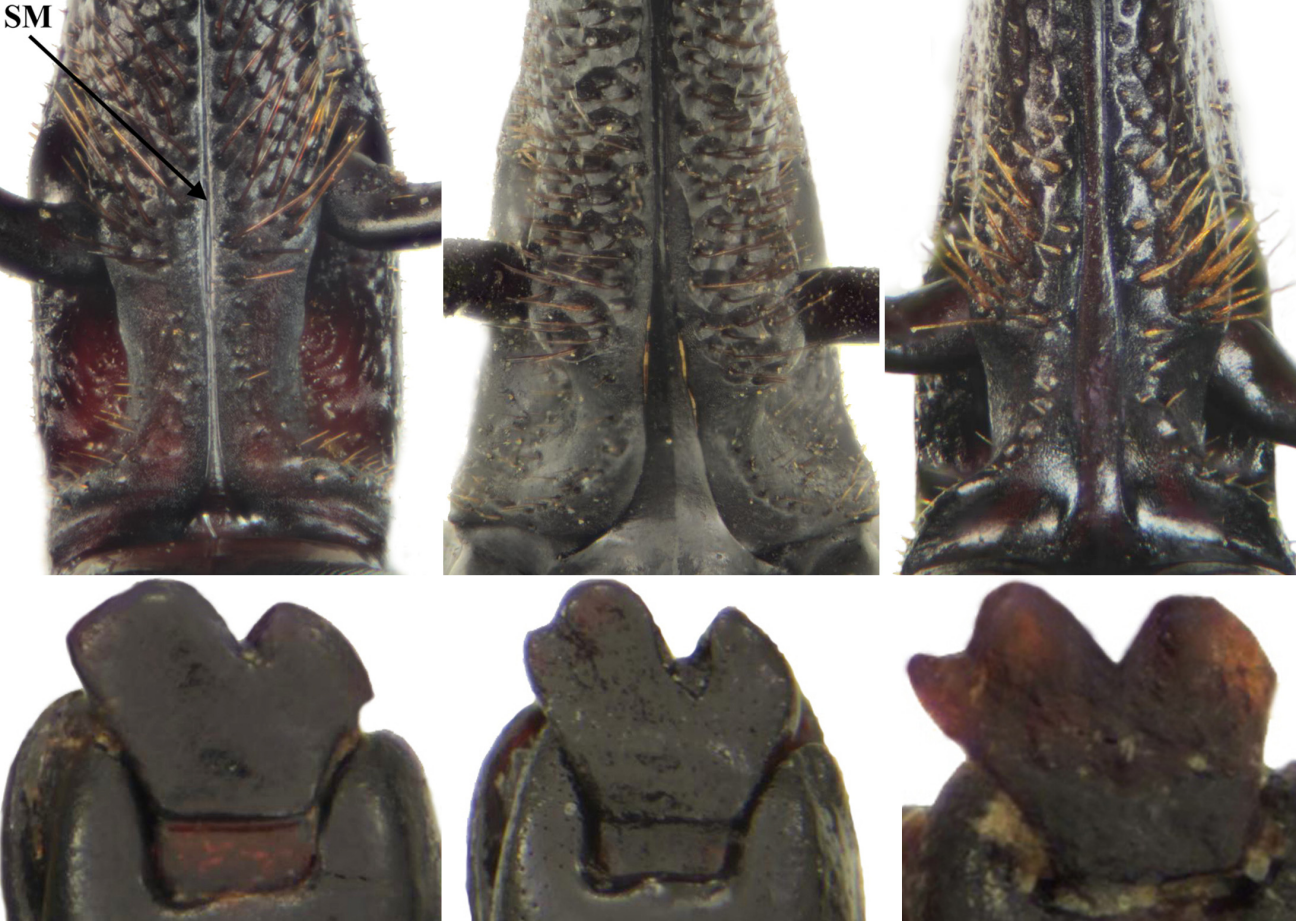
Variation in the subgular suture (SM, top row) and indentation of mandibles (bottom row, lateral view of left mandible) of *Rhynchophorus palmarum* captured in pheromone-baited traps in a sugarcane plantation at CIAT headquarters, Palmira, Colombia.

**Fig 4:**
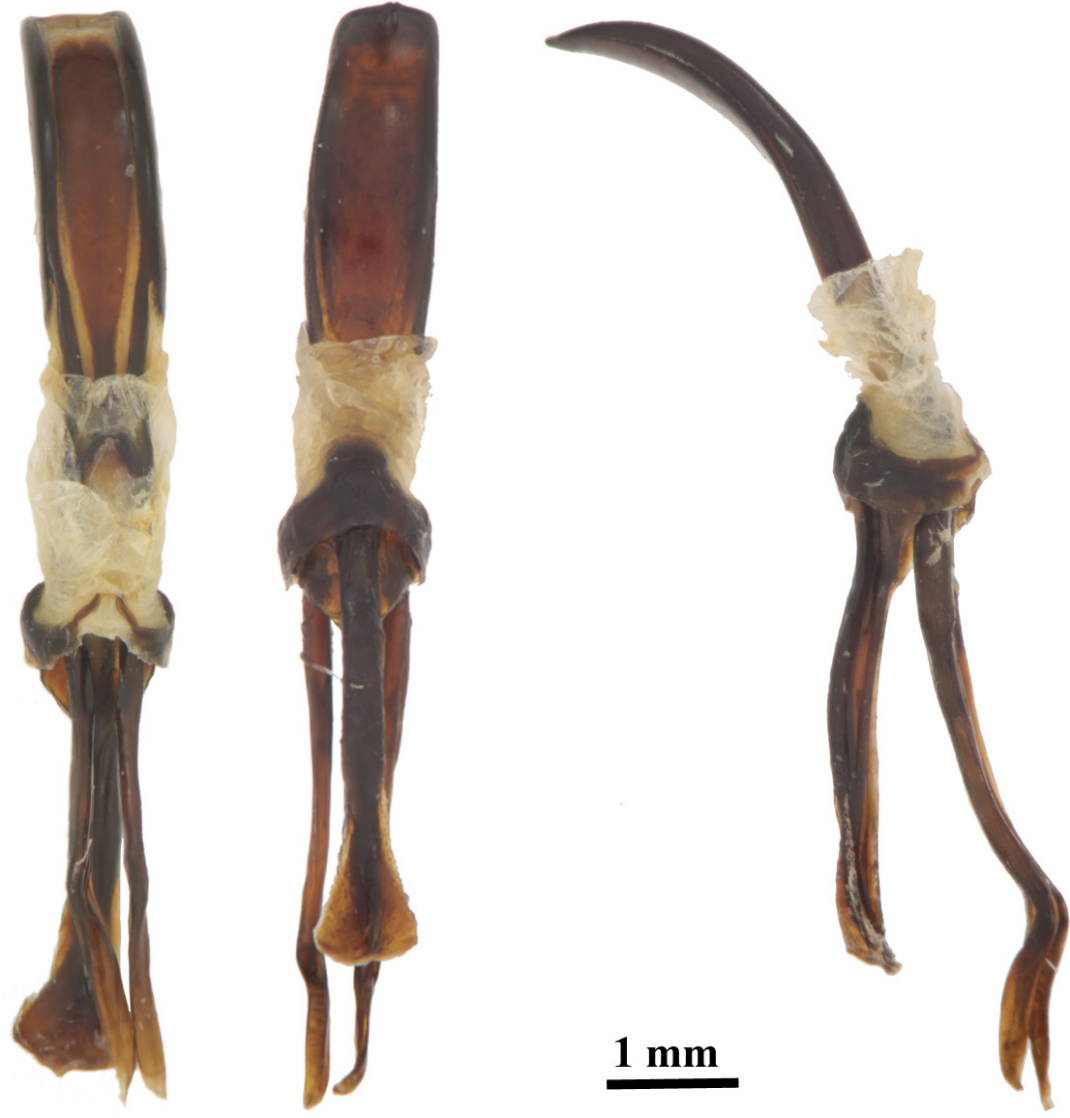
Aedeagus of *Rhynchophorus palmarum* (dorsal (left), ventral (centre), and lateral view (right) captured in pheromone-baited traps in a sugarcane plantation at CIAT headquarters, Palmira, Colombia.

The examination of the morphological characters established by Wattanapongsiri [4] confirmed that all except three specimens captured in the pheromone-baited trap and those preserved in the CEUNP collection presented the characters typical for *R*. *palmarum*. The exceptions were one black and a reddish specimen that presented a wide subgenal suture (0.07–0.08 mm vs. 0.005–0.02 mm in other specimens, n = 184, Fig 3), a character important for differentiating *R*. *palmarum* from the *R*. *ferrugineus* group. A second reddish specimen was found with ratio of the dorsal interocular space to the width of the rostrum at base slightly larger than one third (0.35 vs. 0.16–0.29 in the other specimens, n = 182). The last character studied that is not strictly diagnostic for *R*. *palmarum* but has been used by Giblin-Davis [24] as a useful character, were the shape of the mandibles. Of the 440 specimens studied (eleven specimens were damaged and did not present the mandibles), 159 (black and reddish ones alike) presented tridentate mandibles, while the shape of *R*. *palmarum* mandibles on record is bilobed (Fig 3). The remaining characters conform to those listed for *R*. *palmarum*. We add to the characters provided in [4] measurements for the ventral distance between the antennal scrobes (0.50–2.13 mm, n = 182) and pictures of the aedeagus (Fig 4).

Twenty out of 21 weevil samples successfully amplified the COI sequence. The PCR amplification generated a fragment of approximately 707 bps that was subsequently cloned and sequenced as described above. Three-hundred clones were recovered, 220 were carrying the right size insert (approximately 3 per sample). DNA sequences obtained comprised 235 of the 512 aminoacids that define the COX1 gene of the mitochondrial genome for all samples. The sequences were employed to search the GenBank, EMBL, and BOLD databases to find available sequences with the greatest identity. The information gathered from these three databases allowed the assignment of each weevil sample to a single species.

**Fig 5:**
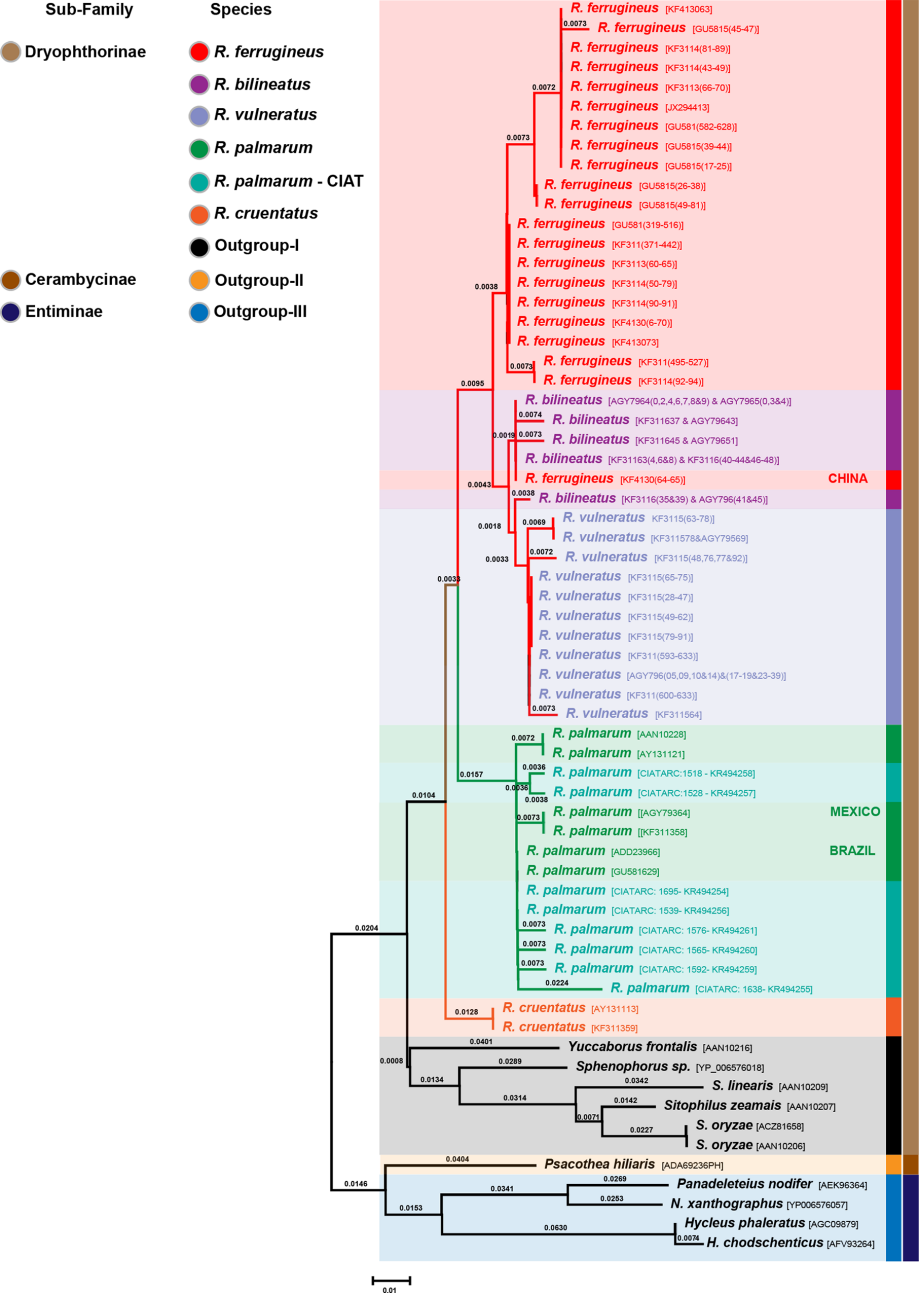
Phylogenetic tree of the palm weevils (genus *Rhynchophorus*). All published and our own sequences were included in the analysis. The African *Rhynchophorus phoenicis* does not appear in the cladogram as the sequences could not be aligned with those of other congeneric species and the similarity did not surpass 84% (reasons unknown).

Twenty consensus DNA sequences of the partial COI region obtained in this study were subjected to phylogenetic analysis, along with 647 related palm weevil COI sequences (460 *R*. *ferrugineus*, 172 *R*. *vulneratus*, *6 R*. *palmarum*, 4 *R*. *bilineatus* 2 *R*. *cruentatus*, 3 *R*. *phoenicis*) retrieved from the GenBank/EMBL/BOLD databases; including six sequences of sub-family Dryophthorinae (genus *Yuccaborus*, *Sphenophorus* and *Sitophilus*), one sequence of sub-family Cerambycinae (genus *Psacothea*) and four sequences of subfamily Entiminae (genus *Pandeleteius*, *Naupactus*, *Hycleus*) were employed as out-group. All 667 COI DNA sequences were translated to aminoacid sequences. The multi-sequence protein alignment revealed that the 632 *R*. *ferrugineus* sequences could be reduced to 29 distinctive haplotypes, while the 20 obtained from our samples corresponded to eight distinctive haplotypes. Hence, a total of 64 sequences were used to build a matrix with pairwise distance values required to obtain a phylogenetic tree with UPGMA (Fig 5), and the agreed tree provided strong support for the morphologically-determined weevil classification. Trees obtained from MEGA are not provided here but can be reconstructed from the original sequence alignment file obtained from CLC main-workbench which is provided as supplementary data (**S2 Database**).

**Fig 6:**
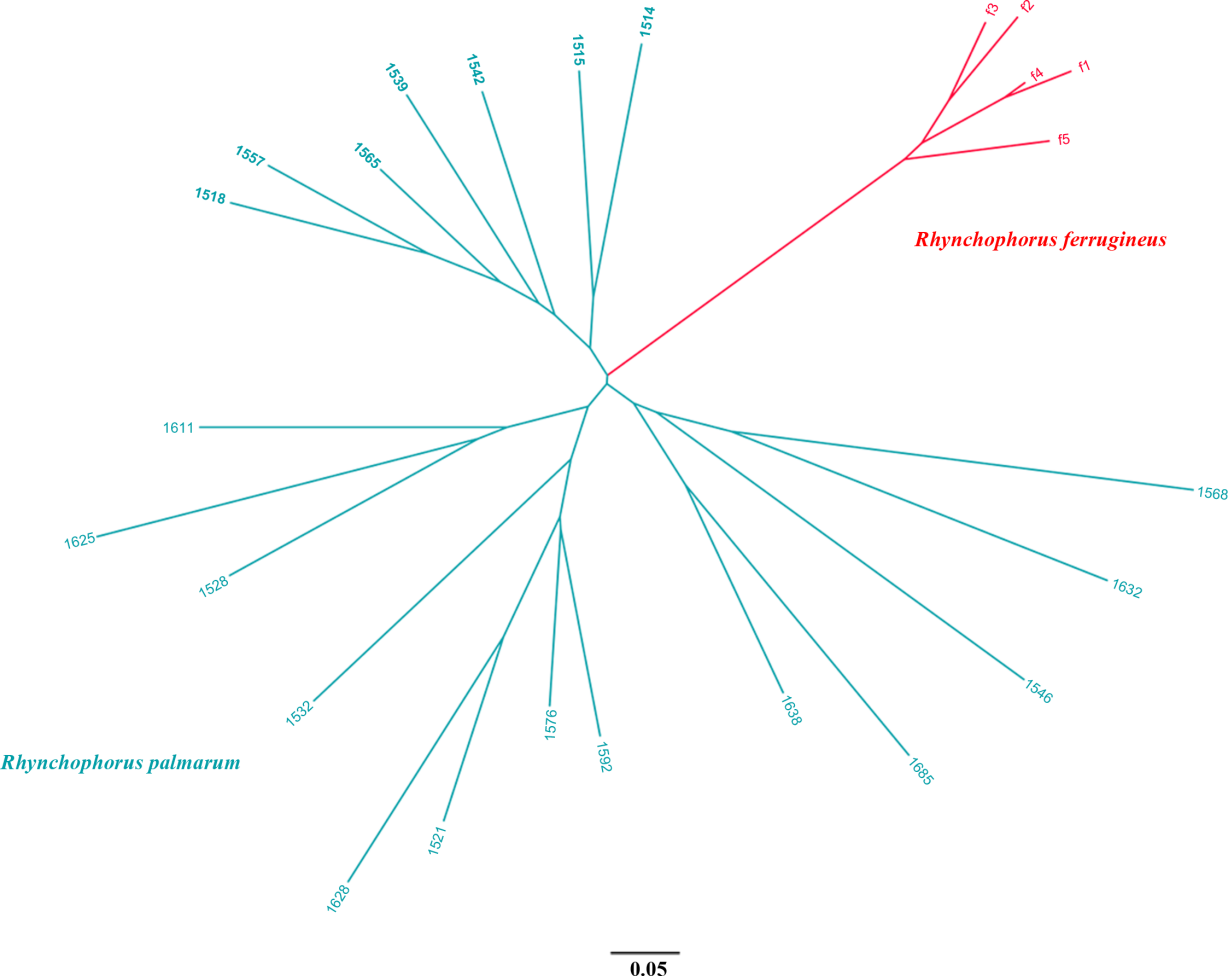
Parental analysis using *Rhynchophorus ferrugineus* specific microsatellites [21] between atypically colored *R*. *palmarum* (collected at CIAT, Palmira, Colombia) and *R*. *ferrugineus* (collected at Oeiras, Portugal).

The analysis of the parental relationship between the 20 *R*. *palmarum* samples and five *R*. *ferrugineus* using micro-satellites of nuclear DNA shows a clear separation of both species (Fig 6). Hence hybridization of both species can be excluded as reason for the red colored *R*. *palmarum*.

## Discussion

Color polymorphism is a common trait in both the animal and plant kingdoms. It is especially frequent in some groups of invertebrates and its biological and ecological significance has been intensively studied over the years. Extensive reviews linked color polymorphism to the process of speciation [25] via apostatic [26] and sexual selection [27]. An additional important aspect of body color is thermo-regulation [28]. These are normally genetically determined and fall into the definition of polymorphism. Variability in body coloration has also been observed as a reaction to habitat [29], color of substrate [30], food quality [31] and crowding [32] and are thus regulated by environmental factors rather than purely genetics and defined as polyphenism [33].

Color polymorphism in arthropods has led to confusion in the taxonomy of a variety of taxa: Cantharidae [34], Dryophthoridae [4, 15], Silphidae [35], (all Coleoptera); Aphidae (reviewed by Lambers [36]), [37] and Cercopidae [38] (all Hemiptera) have been misidentified on the basis of coloration. Mistakes include both failures to distinguish clearly different species because of their similarity in color patterns [39] and the creation of a number of distinct species with no good reason [2, 15, 30]

One such case is known in the genus *Rhynchophorus* where *R*. *ferrugineus* and *R*. *vulneratus* (Panzer) were listed as distinct species on the basis of differences in color and distribution until they were synonymized on the basis of morphological, molecular and breeding data [18]. However, this synonymization is refuted in a recent publication [16].

As Wattanapongsiri [4] states in his revision of palm-boring weevils “The use of color and markings … has caused much confusion in the past”, color variability is common in the genus and in particular *R*. *ferrugineus* is notorious for its many color morphs. Seven different color morphs of *R*. *phoenicis* (F) were registered in a relatively limited sample both in terms of size (174 specimens) and area sampled (a small area in Southwest Province of Cameroon), none of the morphs could be associated to sex [40]. And an almost continuous gradient from weevils with red markings covering most of the body to completely black morphs was observed in a sample of 92 specimens of *Rhynchophorus cruentatus* F. collected from a single palmetto palm in Florida [41]. Color morphs of *R*. *palmarum* were not on record and as the adults remain for days within the cocoon before emerging after the final ecdysis, color change in function of the hardening exoskeleton can also not be an explanation for our observations. It is therefore no surprise that the capture of several specimens resembling *R*. *ferrugineus* led us to believe in an invasion.

To circumvent limitations of morphological characterization in red palm weevil specimens, we amplified Folmer’s COX1 region (i.e. COI) to match DNA samples with morphologically-identified *Rhynchophorus* species. Before undertaking a phylogenetic reconstruction of the red palm weevils in our sample, COI sequences were further examined to characterize for the presence of orthologues or COI-like sequences by looking at the translated amino acid sequence. Moulton [42] suggested incorporating to the barcode methodology the analysis of the derived amino acid sequences particularly when a protein-coding gene region like COI is used. The translation of each COI sequence examined here consistently produced a fragment of 235 amino acids. Our aim to investigate the applicability of DNA barcoding for confirming species identifications of red palm weevil specimens used in this study was successful as 100% match achieved with available weevil entries in GenBank and BOLD databases. In the phylogeny analysis, our eight palm weevil haplotypes fell in the *R*. *palmarum* clade that include specimens found in Brazil and Mexico. Our phylogenetic tree of the genus took into account all published sequences of *Rhynchophorus* and clearly separates *R*. *palmarum* (South and Central America) and *R*. *cruentatus* (Southern USA) from the species original to Asia (*R*. *ferrugineus*, *R*. *bilineatus*, *R*. *vulneratus*). Our findings support the recent resurrection of the latter species name [16]. However, two observations deserve further investigation: two sequences from China labelled *R*. *ferrugineus* (genebank access no. KF4130(64–65)) clearly relate to *R*. *bilineatus*, suggesting a possible failure in the taxonomic identification of the specimens. The sequences published for *R*. *phoenicis* (Africa) (genebank access no. HM043667 and AY131120) do not even appear among the outgroups as the similarity with *Rhynchophorus* sequences is only 84% and the sequences could not be aligned with the remainder of the COI sequences. The cause of this might be a mistake in the registration and the sequences are probably nuclear DNA sequences.

Our results suggest that COI sequence analysis is a very accurate and effective tool for species identification in weevil species. Thus, by comparing molecular markers with diagnostic morphological traits, we provide six new COI sequences that will contribute to build a species-specific sequence library.

The scarcity of the color polymorphism documented from our large collection is confirmed by the single specimen (of 283) in the CEUNP collection, and a photograph of a single specimen from an oil palm plantation in Tumaco, Nariño (1°48'2400"N, 78°45'5300"W) where tens of thousands of weevils were trapped (Gerardo Martínez López, pers comm), and the complete absence of color morphs in the study of Sepúlveda-Cano [15]. The museum record of CEUNP also indicates that this form has been present since at least 1972.

While it is unknown at present how color variation in *R*. *palmarum* may be regulated, apart from genetics, the influence of the environment can also play a major role. Gompel et al. [43] showed that changes in gene regulation during development can influence color expression in *Drosophila* indicating that color polymorphism might be determined by environmental conditions rather than genetic variability.

In addition to the described color polymorphism we found other undocumented morphological variability of *R*. *palmarum* with implications for diagnostics. The more important variation is the occasional presence of a wide subgenal suture (Fig 2), less important the variability in the ratio between the interocular space and width of the rostrum at base. However, both characters are listed in the taxonomic keys [4] to separate *R*. *palmarum* from the *R*. *ferrugineus* group. We therefore recommend to use these characters with special care and apply a combination of all characters cited by Wattanapongsiri [4].

In conclusion, we can state that the feared invasion of the South American continent by the Asian Palm Weevil was a false alarm. Nevertheless, staying alert is important as the problem persists in the Caribbean island of Aruba and Curação.

## Supporting Information

S1 Database Full database of morphological variations of 455 specimens studied. (XLSX)

S2 Database General Rhynchophorus alignment.fa Original alignment of studied *Rhynchophorus* individuals and outgroup taxa. (FA)

S3 Database General Rhynchophorus alignment.txt Original alignment of studied *Rhynchophorus* individuals and outgroup taxa. (TXT)
